# A Case Report and Literature Review of* Clostridium difficile* Negative Antibiotic Associated Hemorrhagic Colitis Caused by* Klebsiella oxytoca*

**DOI:** 10.1155/2018/7264613

**Published:** 2018-09-24

**Authors:** Aaron Fisher, Alexandra Halalau

**Affiliations:** ^1^Internal Medicine Resident, Beaumont Health, Royal Oak, MI, USA; ^2^General Internal Medicine Section Head, Beaumont Health, Royal Oak, MI, USA; ^3^Associate Professor, Oakland University William Beaumont School of Medicine, Rochester, MI, USA

## Abstract

*Klebsiella oxytoca* hemorrhagic colitis is a rare form of antibiotic associated hemorrhagic colitis that is* Clostridium difficile* negative.* Klebsiella oxytoca* colitis has been shown to be triggered by penicillin administration, yet other antibiotics have been implicated as well. It can mimic the appearance of ischemic colitis on endoscopy; however it will generally be found in young, otherwise healthy patients without risk factors. We present a case of a 33-year-old Caucasian female who presented to the emergency room with profuse, bloody diarrhea for 5 days, after a one-week course of ampicillin. Colonoscopy was notable for ulcerated mucosa with erythema and easy friability and the biopsy was suggestive of ischemic colitis. Stool culture was positive for many* Klebsiella oxytoca*. The patient was discharged home with resolution of symptoms after three days in the hospital. She was instructed to avoid penicillin antibiotics and minimize nonsteroidal anti-inflammatory drug use.

## 1. Introduction

Colitis can originate from a wide range of etiologies including ischemic, infectious, and inflammatory bowel disease. A thorough history is imperative to determine the causative nature of the patient's presenting symptoms. Investigations range from observation and stool analysis, to endoscopy with biopsies, depending upon particular risk factors and severity of symptoms. A growing subset of colitis is antibiotic associated colitis. A majority of these cases are secondary to* Clostridium difficile* infection (CDI), with an estimated 453,000 cases of CDI throughout the United States in 2011 [1]. Within the most common antibiotics used, antibiotic associated diarrhea has been reported in 5-10% of patients taking ampicillin, 10 to 25% of those who are treated with amoxicillin–clavulanate, 15 to 20% of those who receive cefixime, and 2 to 5% of those who are treated with other cephalosporins, fluoroquinolones, azithromycin, clarithromycin, erythromycin, and tetracycline [2]. Yet, in cases of clostridium difficile-negative antibiotic associated hemorrhagic colitis (AAHC),* Klebsiella oxytoca* has been isolated at a significantly high rate [3]. In Hogenauer et al.'s study, 385 healthy patients had stool testing for* Klebsiella oxytoca* and in 1.6% of the patients, the pathogen was isolated [4]. Interestingly,* Klebsiella oxytoca* is found mainly in young healthy individuals compared to patients with CDI who are generally older and with previous hospitalizations [4]. Here we present a case of* Clostridium difficile*-negative AAHC caused by* K. oxytoca* infection after taking ampicillin for a urinary tract infection.

## 2. Case Presentation

**Figure 1:**
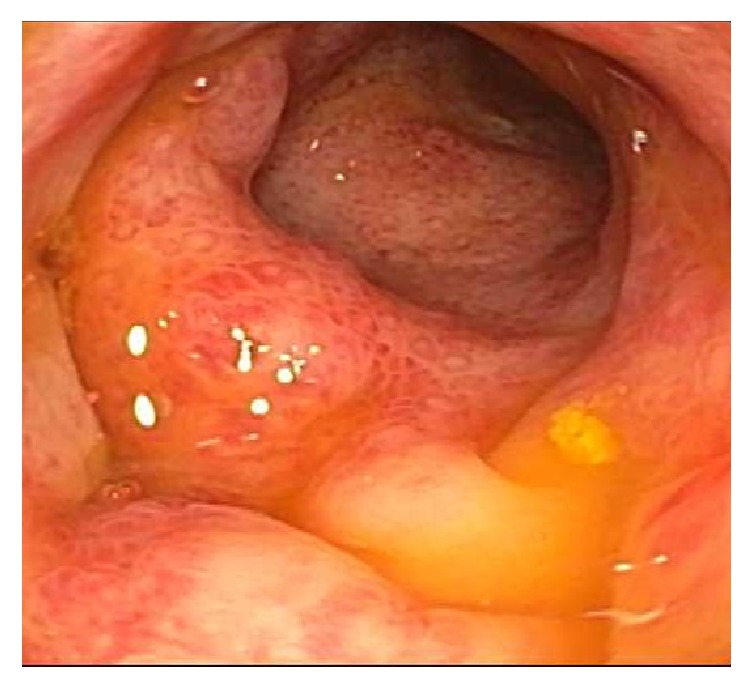
Endoscopic view of ulcerated mucosa with erythema and easy friability, suggestive of moderate colitis.

**Figure 2:**
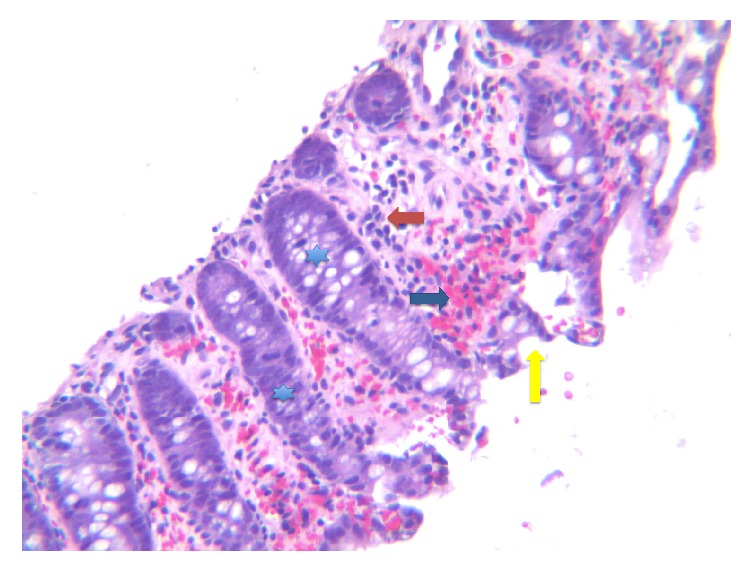
Colonic biopsy. Yellow arrow indicates epithelial surface erosion. Green arrow indicates extravasated red blood cells. Orange arrow indicates lymphocytes. Blue star indicates reactive intestinal glands.

A 33-year-old Caucasian female with significant family history of inflammatory bowel disease (IBD) presents with profuse, bloody diarrhea for 5 days and associated tenesmus and urgency. One day prior to admission, she completed a one-week course of ampicillin for a urinary tract infection and noted that her symptoms began three days after she had initiated treatment. On presentation, patient was hemodynamically stable, afebrile, with mild lower abdominal pain, and a positive guaiac exam. Laboratory findings showed WBC 12.4 bil/L (normal values 3.3–10.7 bil/L), neutrophils 11.0 bil/L (normal values 1.6–7.2 bil/L), Hgb 13.1 g/dL (normal values 12.1–15.0 g/dL), platelets 275 bil/L (normal values 150–400 bil/L), lactic acid 1.4 mmol/L (normal values 0.5–2.2 mmol/L), and liver function tests within normal limits. Initial stool studies that included stool culture, ova and parasite, and* Clostridium difficile* toxin PCR were negative. A colonoscopy was planned as the patient had an extensive family history of IBD and presented with bloody diarrhea.* Klebsiella oxytoca* testing was requested on the stool culture after* Clostridium difficile* PCR came back negative, given her previous use of penicillins. Colonoscopy was notable for ulcerated mucosa with erythema and easy friability, suggestive of moderate colitis throughout the colon with rectosigmoid sparing (Figure 1). Colonic biopsy was remarkable for mucosal congestion and ischemia suggestive of ischemic colitis (Figure 2). Subsequently, requested stool culture was positive for many* Klebsiella oxytoca*. The patient's hematochezia resolved prior to discharge on day 3 of hospitalization, four days after cessation of ampicillin. She was advised to avoid future use of penicillins and minimize nonsteroidal anti-inflammatory drug (NSAID) use.

The patient has continued to follow with her gastroenterologist 10 months after her colonoscopy. She has had epigastric abdominal pain relieved by daily omeprazole. She no longer has documented hematochezia and there has been no repeat colonoscopy.

## 3. Discussion

Colitis is a well-known complication of treatment with antibiotic agents, yet the cause of antibiotic associated hemorrhagic colitis is not completely understood. Several mechanisms have been proposed including allergic reaction, mucosal ischemia, and* Klebsiella oxytoca* infection.* Klebsiella oxytoca* positive hemorrhagic colitis is a rare form of antibiotic associated hemorrhagic colitis that is* Clostridium difficile* negative [4]. In 1978, Toffler et al. were the first to describe AAHC [5]. It has been described that, in a majority of cases, penicillin and penicillin derivative administration typically precede AAHC; however, quinolones and cephalosporins have been described as well [6].

There appears to be a greater incidence of AAHC secondary to* K. oxytoca* in the Japanese population [13].* K. oxytoca* has been reported in the United States as well, yet appears to be more rare. A PubMed literature review was conducted in order to consolidate all English written and adult population case reports on* K. oxytoca* hemorrhagic colitis. Search was conducted via the phrases “Klebsiella oxytoca AND colitis AND case report” and “colitis AND Klebsiella oxytoca.” A total of 74 articles resulted, 18 of which were case reports: 7 written in English, 8 written in French, and 3 written in Japanese. The English written cases range from the years 1999 to 2017 and total 9 cases. Five of the cases are reported from Japan and 4 from cities throughout the United States. Ages range from 37 years to 85 years with 4 males and 5 females. Eight of the 9 cases reported bloody diarrhea. The types of antibiotics reported were three penicillin derivatives (amoxicillin twice and amoxicillin-clavulanic acid once), one macrolide (clarithromycin), three fluoroquinolones (tosufloxacin, enoxacin, and levofloxacin), and one with metronidazole, and one case only reported perioperative antibiotics yet specifics were unknown. Two of the 9 cases noted prior use of a NSAID. Duration of antibiotic use ranged from 1 to 7 days. Interestingly, there was a delay of 3-4 weeks in symptomatology in patients who had taken fluoroquinolones compared to the other patients in which symptoms began closer to time of antibiotic use. Location of endoscopic findings varied throughout the 9 cases. Of the 9 cases, 2 cases were right sided only, 3 cases were from ascending to descending colon, 2 cases only involved the sigmoid colon, 1 case involved the transverse colon, and 1 patient declined colonoscopy. The duration of time for resolution of symptoms ranged from 4 days to 3 weeks. Seven of the 9 cases were treated supportively (one case was treated with prednisolone as well). Two of the 9 cases were treated with fluoroquinolones (one of the cases with metronidazole as well) with no reported recurrence of symptoms. In all cases, antibiotic course was either completed or terminated early prior to symptomatic improvement (Table 1). The diagnosis of* K. oxytoca* can be established by stool [6, 8–10, 12], tissue [7], or bacterial [11] culture. The priority of which culture is of highest yield is uncertain. Of the 9 cases, the most common past medical history was type 2 diabetes mellitus in 3 of the patients, hypertension in 2 of the patients, and systemic lupus erythematosus in 1 of the patients.

Our case is rare given her Caucasian ethnicity; however, as evident by Table 1,* K. oxytoca* in Caucasians have been reported in the past. The patient's symptomatology, duration of symptoms, and time for resolution of symptoms are consistent with previous reported cases [8, 9, 14]. There have been reports of symptoms manifesting weeks after antibiotic course, yet these were reported after fluoroquinolone use [6]. Her age and lack of risk factors for hemorrhagic colitis are also consistent with previous described cases [4]. Colonic biopsy is generally misinterpreted as ischemic colitis [6–9], just as in this case. Diagnosis is made upon stool culture of* K. oxytoca*, which has to be specifically requested. Stool culture of* K. oxytoca* can result within 24 hours of testing. If suspicion is high enough, and physician is aware of such entity, diagnosis of AAHC secondary to* K. oxytoca* can be made and can possibly prevent unnecessary and risky diagnostic procedures. The disease course is generally self-limited with withdrawal of the offending penicillin. Avoidance of NSAIDs is recommended as well, as this can exacerbate the colitis in these patients [7, 14, 15]. It is unclear at this time if readministration of ampicillin would lead to a similar episode [16]; however, the patient has been advised to avoid all penicillins.

In conclusion, we presented a case of* K. oxytoca* colitis in order to raise awareness of the possible etiology of AAHC to be considered in young patients taking antibiotics in which the simple interruption of antibiotic should improve the symptomatology and potentially avoid unnecessary testing or invasive procedures like colonoscopy to be done.

## Figures and Tables

**Table 1:** Literature review of case reports of colitis secondary to *Klebsiella oxytoca*.

**Author**	**Year and Location**	**Patient Age and Gender**	**Presenting symptoms**	**Antibiotic prior to presentation**	**NSAID use prior to presen- tation**	**Duration of Abx prior to sx**	**Endo- scopic location of disease**	**Time to reso- lution of sx**	**Treatment**
Koga et al. [6]	1999 Japan	37 y/o Female	Bloody diarrhea and abdominal pain	Tosu- floxacin tosylate 450 mg	none	5 days∗	Entire colon excluding rectum	2 weeks	Hydration and Loperamide

Koga et al. [6]	1999 Japan	46 y/o Male	Bloody diarrhea and abdominal pain	Enoxacin	none	7 days∗	Right sided	3 weeks	Supportive

Koga et al. [6]	1999 Japan	37 y/o Male	Bloody diarrhea	Levo- floxacin 300 mg	none	7 days∗	Transverse to sigmoid	3 weeks	Supportive

Chen, Cachay, and Hunt [7]	2004 San Diego, CA	79 y/o Male	Diarrhea, abdominal pain, hemato- chezia	none	Aspirin	none	Sigmoid colon	2 weeks	Cipro- floxacin

Philbrick, Ernst [8]	2007 Iowa	63 y/o Male	Watery diarrhea and BRBPR	Amoxicillin 500 mg q8h	Ibuprofen 800mg q8h	5 days	Ascending to descending colon	1 week	Metro- nidazole and Levo- floxacin

Miyauchi, Kinoshita, Tokuda [9]	2013 Japan	67 y/o Female	Mucobloody diarrhea and abdominal pain	Clarithro- mycin 200 mg q12h	none	5 days	Right sided	Not specified	Supportive

Sweetser, Schroede, Pardi [10]	2009 Rochester, MN	67 y/o Female	Watery diarrhea	Peri- operative abx	none	3 days	Sigmoid colon	4 days	Supportive

Kazuyuki et al. [11]	2017 Japan	65 y/o Female	Abdominal pain and hemato- chezia	Amoxicillin 1500 mg and Metro- nidazole 500 mg	none	1 day	Transverse colon	6 days	Bowel rest and prednisolone

Akanbi et al.[12]	2017 Chicago, IL	85 y/o Female	Abdominal pain and muco- bloody diarrhea	Amoxicillin - clavulanic acid	none	5 days	Declined colon- oscopy	5 days	Supportive care

^∗^Symptoms did not start until ~3-4 weeks after antibiotic cessation.

Q8h, every 8 hours; q12h, every 12 hours; BRBPR, bright red blood per rectum; abx, antibiotics; ASA, aspirin; sx, symptoms; y/o, years old.
